# Regulation of Osteoclast Differentiation by Myosin X

**DOI:** 10.1038/s41598-017-07855-9

**Published:** 2017-08-08

**Authors:** Amy Tasca, Kristina Astleford, Ari Lederman, Eric D. Jensen, Beth S. Lee, Rajaram Gopalakrishnan, Kim C. Mansky

**Affiliations:** 1 Department of Diagnostic and Biological Sciences, University of Minnesota, Minneapolis, Minnesota 55455 USA; 2 Department of Physiology and Cell Biology, The Ohio State University, Columbus Ohio, 43210 USA; 3 Department of Developmental and Surgical Sciences, University of Minnesota, Minneapolis, Minnesota 55455 USA

## Abstract

Osteoclasts begin as mononuclear cells that fuse to form multinuclear cells able to resorb bone. The mechanisms that regulate all the steps of osteoclast differentiation are not entirely known. MYO10, an unconventional myosin, has previously been shown in mature osteoclasts to play a role in attachment and podosome positioning. We determined that MYO10 is also expressed early during osteoclast differentiation. Loss of MYO10 expression in osteoclast precursors inhibits the ability of mononuclear osteoclasts to fuse into multinuclear osteoclasts. Expression of *Nfatc1*, *Dc-stamp*, *Ctsk*, and *β*_3_ *integrin* is reduced in the osteoclasts with reduced MYO10 expression. A slight reduction in the osteoclasts ability to migrate, as well as a reduction in SMAD 1/5/8 phosphorylation are also noted with reduced MYO10 expression. Interestingly we also detected a change in the ability of the osteoclast precursors to form tunneling nanotubes (TNTs), which suggests that MYO10 may regulate the presence of TNTs through its interaction with the cytoskeletal proteins.

## Introduction

Osteoclasts, which are large multinucleated cells formed from the fusion of multiple mononuclear precursors^1^, are the primary resorptive cells of the skeleton. They facilitate the removal of old bone and aid in maintaining mineral homeostasis^2^. Osteoclast differentiation, including fusion of mononuclear osteoclasts, is regulated by two cytokines: macrophage colony stimulating factor (M-CSF) and receptor activator of NF-κB ligand (RANKL). Fusion is a genetically programmed process that can be divided into three phases: competence (differentiation); commitment (migration & adhesion); and cell fusion (membrane merging & cytoplasmic mixing)^3^. In order for osteoclast fusion to occur precursors must first be recruited and migrate to the bone cell surface; gene expression must be altered to establish a fusion-competent status; cell-cell recognition and attachment must occur; finally, fusion and cellular reorganization takes place in order to form active multinucleated osteoclasts^4^. Identification of dendritic cell-specific transmembrane protein (DC-STAMP) and discovery that it is highly expressed in multinucleated osteoclasts but not in mononuclear precursors was crucial to our limited understanding of how osteoclasts fuse^5^. Although basic principles for osteoclast fusion are understood, the precise mechanism, sequence of events, and factors involved in osteoclast fusion still remain unclear.

Myosins are actin-based molecular motors that utilize ATP to perform many cellular functions. Myosin X (MYO10) is an unconventional myosin. It is essential for formation of filopodia, which are slender actin-based extensions in cells^6^. MYO10 has also been implicated in playing a role in cell adhesion^7^. It has been shown that MYO10 is required for attachment and forming the sealing zone in mature osteoclasts^8^. However, the role of MYO10 in regulating osteoclast differentiation is unknown. The goal of the current study is to determine the role of MYO10 in the early stages of osteoclast differentiation and fusion. We hypothesize that MYO10 is a key factor involved with osteoclast differentiation. Osteoclast precursors with reduced levels of MYO10 expression remain mononuclear and unable to fuse and differentiate into multinuclear cells. Furthermore, we determined that MYO10 regulates osteoclast migration, tunneling nanotube formation and actin organization necessary for osteoclast fusion.

## Results

### MYO10 is expressed during early stages of osteoclast differentiation

**Figure 1:**
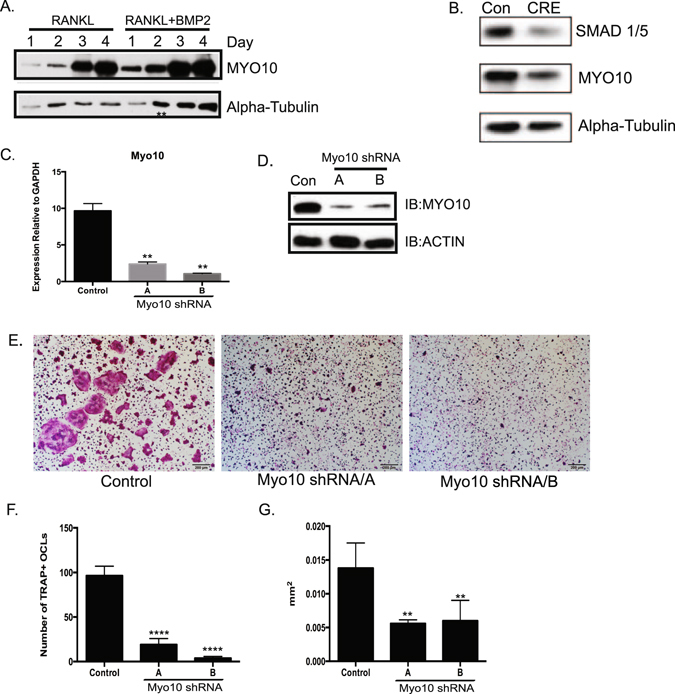
Myo10 expression is required for osteoclast differentiation. (**A**) Western blot of osteoclast lysates treated with M-CSF and RANKL (10 ng/mL, left lanes) or M-CSF, RANKL (10 ng/mL) and BMP2 (200 ng/mL, right lanes) for various days. MYO10 and alpha-tubulin expression was analyzed. (**B**) BMMs were cultured from SMAD1/5 floxed mice and infected with a control or CRE expressing adenovirus. Osteoclasts were treated with M-CSF and RANKL for 3 days. The lysates were analyzed for expression for SMAD1/5 and MYO10 by Western blot. (**C**) BMMs were cultured from C57Bl/6 mice and infected with lentivirus expressing either a control shRNA or one *Myo10* targeting shRNA. Real time RT-PCR was used to measure *Myo10* gene expression following 48 hours of infection by lentivirus (**D**) MYO10 protein levels in shRNA-treated cells were analyzed by western blot (**E**–**G**) BMMs were differentiated in the presence of M-CSF and RANKL, TRAP stained, imaged and quantified for number and cell area. Only cells with 3 or more nuclei were quantified. Western blots were cropped to show only relevant bands. Experiments were done at least three times and values represent the mean ± SD. **p < 0.01, ****p < 0.0001.

We previously demonstrated that osteoclasts treated with BMP2 have enhanced RANKL-dependent osteoclast differentiation^9, 10^. Furthermore, in BMP2 treated osteoclast cultures, the enhancement of osteoclast differentiation is not due to changes in the rate of proliferation or apoptosis^9^. To determine potential mechanisms by which BMP2 enhances osteoclast differentiation, we began to identify genes differentially regulated by BMP2 treatment of osteoclasts. In endothelial cells MYO10 had been previously shown to be a target of BMP6^11^. MYO10 is known to play a role in sealing zone patterning in osteoclast resorption^8^ but it is not known if MYO10 is expressed or plays a role in early stages of osteoclast differentiation. To determine whether MYO10 is expressed during early stages of osteoclast differentiation, protein lysates from different days of RANKL- or RANKL- and BMP2-treated osteoclast cultures were analyzed by Western blot. As shown in the left panel of Fig. 1A, we detected a weak band of MYO10 expression at one day with RANKL treatment and a more intense band after one day of BMP2 and RANKL treatment of osteoclast cultures. This induction continued even after two days of BMP2 treatment leading to increased BMP2-mediated expression throughout osteoclast differentiation (Fig. 1A, Supplemental Figure S3A).

BMP signaling can occur through a canonical, SMAD 1/5/8, pathway. Using mice that are floxed for *Smad 1* and *Smad 5*, we isolated bone marrow macrophages (BMMs) from *Smad1* ^*fl/fl*^; *Smad5* ^*fl/fl*^ mice and infected with either a control or CRE expressing adenovirus. We have previously shown that this experimental system creates SMAD 1/5-deficient osteoclasts^12^. When we analyzed osteoclasts that had reduced SMAD1/5 expression, we also detected decreased MYO10 expression (Fig. 1B, Supplemental Figure S3B). This data suggests that MYO10 expression is at least in part regulated by the BMP canonical signaling pathway.

### MYO10 expression is required for osteoclast differentiation and activity

As we were able to detect MYO10 expression during early stages of osteoclast differentiation, we wanted to establish the importance of MYO10 in this process. Osteoclast precursors from WT mice were infected with either a lentivirus expressing a scrambled (control) shRNA or lentiviruses expressing one of two distinct shRNAs targeting *MYO10*. Real time PCR was used to measure *Myo10* expression 48 hours after infection by the lentivirus (Fig. 1C). We detected a significant reduction (approximately 4–5 fold) in *Myo10* in the shRNA-infected cells compared to the control-infected cells. Correspondingly, MYO10 protein levels were also reduced in cultures infected with *Myo10* shRNA compared to the cells infected with the control shRNA (Fig. 1D, Supplemental Fig. 3C).

To determine the functional significance of the loss of *MYO10* expression on osteoclast differentiation, BMMs were infected with either a control lentivirus or a lentivirus expressing a *Myo10* shRNA and differentiated in the presence of M-CSF and RANKL for 5 days. Cultures were fixed, stained for TRAP (Fig. 1E), and quantified by counting number of cells with 3 or more nuclei (Fig. 1F) or measuring size of multinuclear cells (Fig. 1G). Osteoclasts with reduced MYO10 expression were five fold less numerous and two fold smaller in area compared to the controls. While our data suggests that MYO10 expression is responsive to BMP2 treatment of osteoclasts, we were unable to rescue osteoclast differentiation of *Myo10*-deficient cells with increasing doses of BMP2 (data not shown).

To understand if reduced *MYO10* expression leads to changes in osteoclast activity, we cultured BMMs on calcium phosphate coated plates and measured pit number, average pit size and percent area of resorption. As shown in representative images of the calcium phosphate wells containing cells infected with *Myo10* shRNA (Supplemental Figure S1A), there was at least a ten-fold reduction in resorption pits (Supplemental Figure S1B) and percent area demineralized (Supplemental Figure S1D) by osteoclasts with reduced *MYO10* expressed compared to the control infected cells. However, there was no significant difference in the average size of the resorption pit between control osteoclasts and osteoclasts with reduced *MYO10* expression (Supplemental Figure S1C).

**Figure 2:**
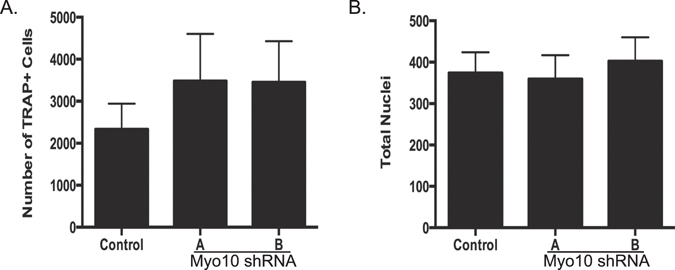
Reduction of Myo10 expression does not affect number of mononuclear osteoclast precursors. BMMs were cultured from C57Bl/6 mice and infected with lentivirus expressing either a control shRNA or one of two *Myo10* targeting shRNA. BMMs were differentiated in the presence of M-CSF and RANKL for 2 days. (**A**) Quantification of day 2 TRAP-stained cells. (**B**) Quantification of nuclei of day 2 DAPI stained cells. Experiments were done at least three times and values represent the mean ± SD.

To determine whether the decrease in osteoclast number, size and activity was due to a reduction in TRAP positive osteoclast precursors capable of fusing to form multinuclear cells, we counted the number of TRAP positive cells in culture (Fig. 2A) and the total number of nuclei present (Fig. 2B) after RANKL stimulation but prior to the onset of osteoclast fusion. We found that there is an equal or greater number of mononuclear TRAP positive preosteoclasts present in cultures where MYO10 is reduced. These results demonstrate that lack of multinuclear cells in *Myo10* shRNA expressing osteoclasts is not due to fewer TRAP positive mononuclear cells but the inability of these mononuclear cells to proceed through differentiation including but not limited to the steps necessary for fusion.

### Osteoclast fusion and migration are reduced when MYO10 expression is reduced

**Figure 3:**
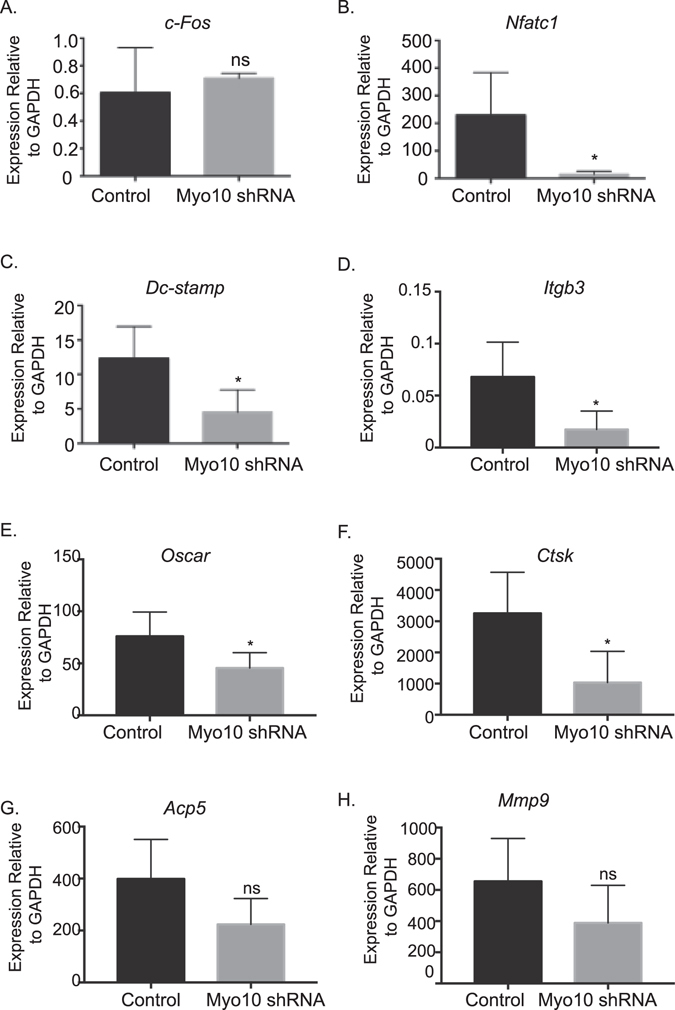
Reduction in MYO10 alters key osteoclast gene expression. Real time RT-PCR was used to measure gene expression following 48 hours of infection by lentivirus of (**A**) *c-Fos* (**B**) *Nfatc1* (**C**) *Dc-stamp* (**D**) *Itgb3* *-* (**E**) *Oscar* (**F**) *Ctsk* (**G**) *Acp5* and (**H**) *Mmp9*. Experiments were performed at least three times and values represent the mean ± SD. *p < 0.05.

To further determine the mechanism of the decreased osteoclast multinucleation that we observed in Fig. 1, changes in osteoclast gene expression were assessed. In the *Myo10* shRNA infected cells, we measured no change in *c-Fos* (Fig. 3A) expression. There was a sixteen fold reduction in the expression of *Nfatc1* (Fig. 3B), three fold reduction in *Dc-stamp* (Fig. 3C), four fold reduction in *Itgb3* *-* (Fig. 3D), one and a half fold reduction in *Oscar* (Fig. 3E), and a three fold reduction in *Ctsk* (Fig. 3F) in the *Myo10* shRNA infected cells compared to the control infected cells. There was a reduction in *Acp5* and *Mmp9* expression in *Myo10*-deficient osteoclasts; however, it was not significant (Fig. 3G,H). Given that *Dc-stamp* expression was significantly reduced when *Myo10* expression is reduced, one possible mechanism by which osteoclast fusion in *Myo10* shRNA expressing cells is inhibited is through changes in *Nfatc1* and *Dc-stamp* expression.

**Figure 4:**
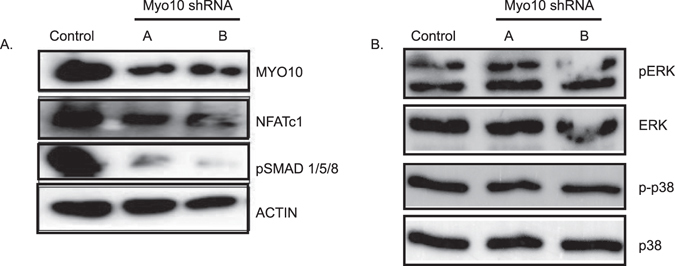
Myo10-deficient osteoclasts undergo less pSMAD1, 5, 8 activation. Western blot of osteoclast extracts that were cultured in the presence of M-CSF and RANKL for 3 days. (**A**) MYO10, NFATc1, and pSMAD 1/5/8 levels were analyzed by Western blot. Actin was analyzed as a loading control. (**B**) pERK, ERK, p-p38 and p38 levels were analyzed by Western blot. Western blots were cropped to show only relevant bands. Full length western blots are presented in Supplemental Fig. 4.

In endothethial cells, loss of MYO10 expression has been shown to regulate SMAD 1/5/8 activation by BMP6^11^. Our lab has demonstrated that BMP stimulation enhances osteoclast differentiation^9, 10, 12, 13^. To determine if similar to endothelial cells, osteoclasts deficient in MYO10 expression have altered pSMAD 1/5/8 signaling, extracts of osteoclast lysates were analyzed by Western blot for changes in BMP signaling pathways in control or *Myo10*-deficient osteoclasts. We did detect a reduction in phosphorylated SMAD1/5/8 in Myo10-shRNA expressing osteoclasts (Fig. 4A, Supplemental Fig. 4A); however, we did not detect any changes in the MAPK signaling pathways, ERK (p42/44) or p38, which are part of the BMP noncanonical signaling pathway (Fig. 4B, Supplemental Fig. 4B). This data suggests one other possibility for osteoclasts with reduced MYO10 expression may be the inability to differentiate due to changes in SMAD 1/5/8 phosphorylation. Lastly we detected a reduction in NFATc1 expression in MYO10-deficient osteoclasts (Fig. 4A) by Western blot which correlates with our qRT-PCR data presented in Fig. 3.

**Figure 5:**
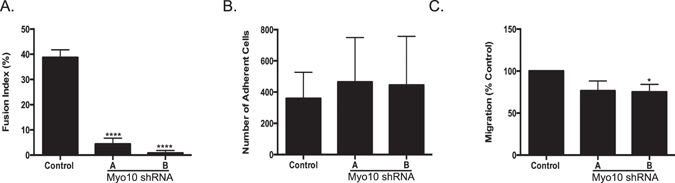
Osteoclast fusion and migration are reduced when MYO10 expression is reduced. (**A**) Fusion index was calculated for day three osteoclasts using the formula fusion index (%) = total number of nuclei within giant cells/total number of nuclei counted × 100. (**B**) Adhesion of day two primary osteoclasts to vitronectin coated plates. (**C**) Transwell migration of day two primary osteoclasts. Experiments were performed at least three times and values represent the mean ± SD. *p < 0.05, ****p < 0.0001.

Another step in the osteoclast differentiation process is adhesion and migration. We next looked at fusion, adhesion, and migration in the *Myo10* shRNA infected osteoclasts. We first calculated the fusion index (Fig. 5A) and determined that it was significantly reduced in osteoclast cultures that had been infected with *Myo10* shRNA compared with the controls. Since MYO10 has been implicated in playing a role in adhesion in various cell types^7, 8^, we tested the ability of preosteoclasts to adhere to vitronectin-coated plates (Fig. 5B). There was no significant difference in the osteoclasts’ ability to adhere when comparing the *Myo10* shRNA infected cells and the control infected cells. Cells must also be able to migrate and find one another in order to fuse. Using a trans-well migration assay (Fig. 5C), we determined that there was a significant but modest decrease in cell migration in the *Myo10* shRNA infected cells compared to the control infected cells.

### Reduction in MYO10 expression results in osteoclast cytoskeletal defects

**Figure 6:**
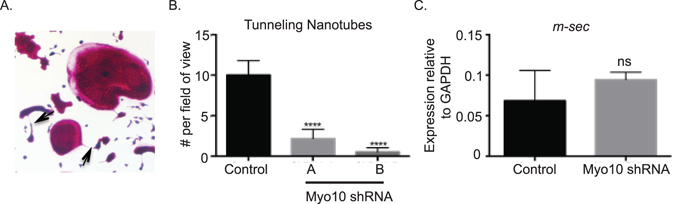
Tunneling nanotube formation is reduced when MYO10 expression is reduced. (**A**) TRAP image indicating presence of tunneling nanotube. (**B**) Quantification of tunneling nanotubes at day 2 of culture present as viewed on the 4x objective. (**C**) RT-PCR quantification of *m-sec* gene expression of day 2 primary osteoclasts.

Cellular reorganization must occur for fully functional multinuclear osteoclasts to form. Tunneling membrane nanotubes (TNTs) are intercellular bridges that have been shown to form between TRAP-positive osteoclasts (Fig. 6A and Supplemental Figure S2, as indicated by the arrows)^14^. It has been shown that TNTs in osteoclasts are formed with RANKL stimulation and contain both actin filaments as well as β-tubulin^14^. As MYO10 is a key regulator of TNT formation in neuronal cells^15^, TNT formation was measured in the *Myo10* shRNA infected osteoclast cultures (Fig. 6B). We determined that TNT formation is significantly diminished when MYO10 expression is reduced in osteoclasts (Fig. 6B). TNT formation in osteoclasts is accompanied by significant induction of the *m-sec* gene, and loss of *m-sec* expression leads to a significant reduction in TNT forming and TRAP positive multinuclear cells^14^. However, *m-sec* gene expression is not significantly changed when MYO10 expression is reduced in osteoclasts (Fig. 6C). These data suggest that MYO10 regulates TNT formation in osteoclasts independent of *m-sec* expression.

**Figure 7:**
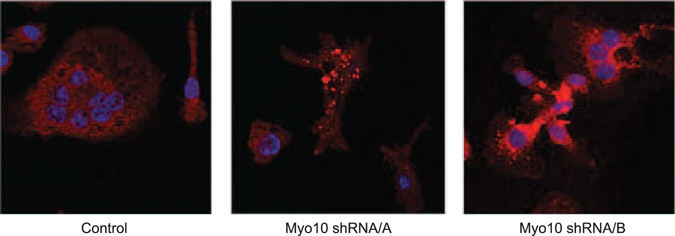
The actin cytoskeleton is altered when MYO10 expression is reduced. BMMs were flushed from C57Bl/6 mice and grown on glass coverslips. The cells were infected with lentivirus expressing either a control shRNA or *Myo10* targeting shRNA and grown in the presence of M-CSF and RANKL. On day 3 cells were fixed and stained for actin (red) and nuclear DNA (blue) and imaged using confocal microscopy.

The actin-signaling network has been shown to regulate osteoclast multinucleation^16, 17^. Wang *et al*. demonstrated that bands of actin filaments, which were involved in cell fusion, originated in the cell bodies and extended into fusopods. Fusopods express MYO10 at their tips. To determine if an additional mechanism by which osteoclasts expressing less MYO10 were not fusing was due to change in their actin organization, we completed immunofluorescence on cells with MYO10 knockdown (Fig. 7) for 3 days. We analyzed cells at 3 days after RANKL treatment so as to compare cells that have been knocked down for MYO10 at the fusion stage of osteoclast differentiation. At this stage, we would not expect control infected cells to have formed an actin ring normally detected in mature multinuclear osteoclasts. Compared to control shRNA infected osteoclasts which show actin staining throughout the cell, *Myo10* shRNA expressing osteoclasts show punctate or globular masses of actin. These data further demonstrate that MYO10 deficiency alters actin reorganization in differentiating osteoclasts.

## Discussion

The mechanisms that regulate the early stages of osteoclast differentiation and fusion are still largely unknown. This study provides compelling evidence that MYO10 expression is necessary for migration of osteoclast precursors during the fusion process. Osteoclasts that express reduced levels of MYO10 are unable to fuse into multinuclear cells and demineralize a calcium phosphate substrate. Osteoclasts expressing *Myo10* shRNAs were reduced in their ability to migrate, formed fewer TNTs and displayed a disorganized actin network compared to control infected osteoclasts. These data suggest that expression of MYO10 in osteoclasts is necessary for the movement of mononuclear osteoclasts towards each other to allow for fusion to occur.

TNTs are intercellular bridges that have been shown to have an important role in cell-cell communication for immune cells such as NK cells, dendritic cells and macrophages^14^. In osteoclasts, TNTs are induced by RANKL and contain actin filaments as well as microtubules. Takahashi *et al*. demonstrated that cell surface molecules such as DC-STAMP are able to migrate and penetrate other precursors through TNTs^14^. Previously McMichael *et al*. demonstrated that MYO10 could interact with β-tubulin and was involved in podosome and sealing zone positioning in mature osteoclasts^8^. They also demonstrated that MYO10, through its microtubule-binding MyTH4 tail domain, transitions osteoclast adhesion structures from clusters to podosome belts and sealing zones^8^. It may be that MYO10 uses its MyTH4 tail domain to interact with microtubules to aid in the formation of the TNTs.

To our surprise, we did not see differences in adhesion between control and *Myo10* knockdown osteoclasts. It was previously reported that overexpression of the tail or the FERM domain of MYO10 disrupted the interaction between endogenous MYO10 and integrins, and the ability of M21 cells to adhere to a vitronectin-coated surface^7^. Future studies will focus on whether MYO10 interacts with integrins or other FERM-containing proteins such as talins, which are necessary for osteoclast activity^18^.

Yagi *et al*. demonstrated that *Dc-stamp* expression was necessary for osteoclast fusion. Our results support this finding. *Dc-stamp* expression was significantly reduced when MYO10 expression was inhibited. The reduction in *Dc-stamp* may be due to decreased levels of *Nfatc1*, which has been shown to regulate *Dc-stamp* expression^19^. However, the question is then raised as to why there is a reduction in *Nfatc1* expression since MYO10 is not a transcription factor and should not be able to directly regulate the expression of *Nfatc1*. In endothelial cells, MYO10 has been shown to participate in an amplification loop of BMP signaling^11^. More recently BMP signaling in osteoblasts has been shown to regulate NFATc1 nuclear localization through activation of canonical SMAD signaling^20^. Data presented in this study demonstrates that decreased MYO10 expression leads to a decrease in the levels of detected phosphorylated SMAD1, 5, 8. Preliminary data from our lab has demonstrated that loss of SMAD signaling in osteoclasts results in less NFATc1 in the nucleus of osteoclasts (private communication K. Mansky). Therefore, it is possible that MYO10 through BMP signaling pathways may be regulating NFATc1 expression or localization. This hypothesis will be tested in future experiments.

**Figure 8:**
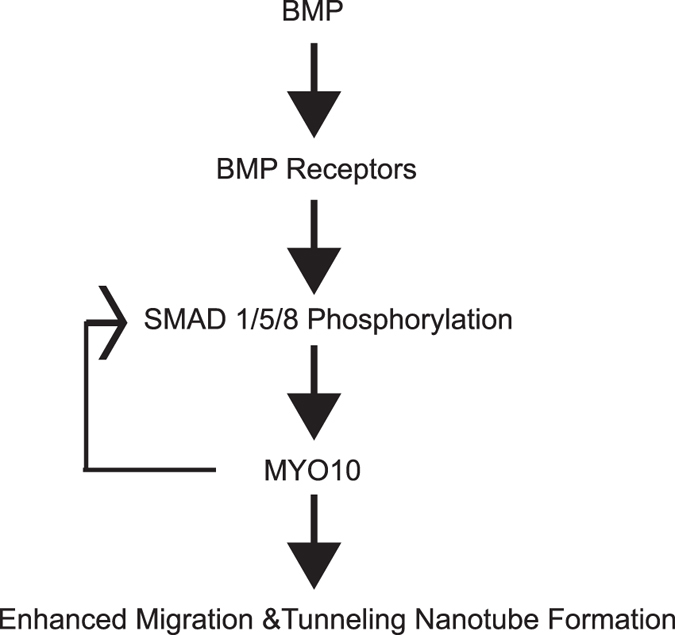
Mechanism of MYO10 regulation of osteoclast differentiation. Illustration of a pathway of MYO10 regulation of osteoclast differentiation.

Our findings suggest that MYO10 is expressed during early stages of osteoclast differentiation. While McMichael *et al*. demonstrated that MYO10 is necessary for osteoclast activity^8^, our data further demonstrate that MYO10 expression is required for osteoclast differentiation. Based on our data we propose that BMP canonical signaling regulates MYO10 expression which in turn regulates preosteoclast migration and tunneling nanotube formation. In turn MYO10 by yet an unknown mechanism regulates SMAD 1/5/8 activation and either NFATc1 expression and/or localization (Fig. 8). Additionally, MYO10 plays a crucial role in developing proper actin substructure and cell motility. Therefore, we conclude MYO10 regulates osteoclast differentiation.

## Materials and Methods

### Ethics

All experiments were performed in accordance with institutional guidelines by the Institutional Animal Care (IACUC) and the Committee of the Office for the Protection of Research Subjects at the University of Minnesota, Minneapolis. Lentiviral generation and viral transduction of osteoclasts were performed under approval of the University of Minnesota Institutional Biosafety Committee (IBC). All experimental protocols were approved by both IACUC (protocol #1507-32820A) and IBC (protocol #1506-32679H) at the University of Minnesota, Minneapolis.

### Harvesting of bone marrow/Primary OCLs

Primary bone marrow macrophages were harvested from the femurs and tibiae of 4-week-old C57Bl/6 mice. The femurs and tibiae were dissected and adherent tissue was removed. The ends of the bones were cut and the marrow was flushed from the inner compartments. Red blood cells were lysed from the flushed bone marrow tissue with RBC lysis buffer (150 mM NH_4_Cl, 10 mM KHCO_3_, 0.1 mM EDTA, pH7.4) and the remaining cells were plated on 100 mm plates and cultured overnight in osteoclast medium (phenol red-free Alpha-MEM (Gibco) with 5% fetal bovine serum (Hyclone), 25 units/mL penicillin/streptomycin (Invitrogen), 400 mM L-Glutamine (Invitrogen), and supplemented with 1% CMG 14–12 culture supernatant containing M-CSF. The non-adherent cell population, including osteoclast precursor cells, was then carefully separated and re-plated at approximately 2 × 10^5^ cells/cm^2^ in a 12 well plate with osteoclast medium supplemented with 1% CMG 14–12 culture supernatant containing M-CSF. Two days later, this medium was replaced with medium containing 1% CMG 14–12 culture supernatant and 10 ng/mL RANKL (R&D Systems) to stimulate osteoclast differentiation.

### Lentiviral Transfection

Bone marrow macrophages were isolated as described above. Prior to stimulation with RANKL, the cells were incubated with lentivirus expressing either a control shRNA (Open Biosystems) or a *Myo10* shRNA (Open Biosystems, #508495 and #508491) at 37 °C in the presence of M-CSF. After 24 hours, medium containing the lentivirus was removed and cells were fed with 1.0% CMG 14–12 culture supernatant and RANKL (10 ng/ml). After two days, RNA was extracted for use in qRT-PCR or protein was extracted for western blotting. For every experiment analyzed, RNA was collected and extracted to determine that sufficient knockdown was achieved. On day five cells were fixed in 4% paraformaldehyde (PFA) and stained for TRAP.

### Harvesting RNA

Quantitative real-time PCR was performed using the MyiQ Single Color Real-Time PCR Detection System (Biorad). RNA was harvested from cells using Trizol Reagent (Ambion, Life Technologies) and quantified using UV spectroscopy. cDNA was prepared from 1 µg RNA using the iScript cDNA Synthesis Kit (Biorad) as per the manufacturer’s protocol. Experimental genes were normalized to *Gapdh. Gapdh* (Forward) 5′-TGCACCACCAACTGCTTA; (Reverse) 5′-GATGCAGGGATGATGTTC; *Nfatc1* (Forward) 5′-TCATCCTGT CCAACACCAAA (Reverse) 5′-TCACCCTGGTGTTCTTCCTC; *Dc-stamp* (Forward) 5′-CAGACTCCCAAATGCTGGAT (Reverse) 5′-CTTGTGGAGGAA CCTAAGCG; *Myo10* (Forward) 5′-CCTGCCCATAGTCTGTCTGG (Reverse) 5′-CAATGGACAGCTTCTTTCCC; *M-Sec* (Forward) 5′-GTGCAGAACCTCTACCCCAATG (Reverse) 5′-TGGAGAATGTCGATGGCCCA; *c-fos* (Forward) 5′-CCAAGCGGAGACAGATCAACT; *c-fos* (Reverse) 5′-TCC AGTTTTTCCTTCTCTTTCAGCAGA; *Itgb3* (Forward) 5′-CTGGTAAAACGC GTGAAT; (Reverse) 5′-CGGTCATGAATGGTGATGAG; *Oscar* (Forward) 5′-TCATCTGCTTGGGCATCATA; (Reverse) 5′-ACAAGCCTGACAGTGTGGTG; *Ctsk* (Forward) 5′-AGGGAAGCAAGCACTGGATA; (Reverse) 5′-GCTGGCTGG AATCACATCTT; *Acp5* (Forward) 5′-CGTCTCTGCACAGATTGCA; (Reverse) 5′-GAGTTGCCACACAGCATCAC; *Mmp9* (Forward) 5′-GTTTTTGATGCTATTGCT GAGATCCA; (Reverse) 5′-CCCACATTTGACGTCCAGAGAAGAA.

### Immunoblotting

Cell protein lysates were harvested from osteoclasts in modified RIPA buffer (50 mM Tris pH 7.4, 150 mM NaCl, 1% IGEPAL, 0.25% sodium deoxycholate, 1 mM EDTA) supplemented with Halt Protease & Phosphatase Inhibitor Cocktail (Thermo Scientific). Lysates were cleared by centrifugation at 12,000 × *g* at 4 °C. Proteins were resolved by SDS-PAGE and transferred to PVDF membrane (Millipore). MYO10 antibody (HPA024223) antibody was obtained from Sigma Life Science. pSMAD1/5/8 (13820), pERK (9101), p-p38 (9211), p38 (9212) and alpha-tubulin (2144) antibodies were obtained from Cell Signaling. NFATc1 (sc-7294) was obtained from Santa Cruz. HRP-conjugated anti-rabbit was incubated with membranes, washed, and incubated with Advansta-Western Bright Sirius detection agent. Images were acquired on Chemitouch (BioRad) or on film (Kodak) and cropped in Adobe Photoshop.

### TRAP Stain

Primary osteoclasts were fixed with 4% paraformaldehyde (PFA) and washed with PBS. The cells were then stained for tartrate resistant acid phosphatase (TRAP) expression as previously described^12^. Cells were then observed and captured with light microscopy and the measurements were analyzed using NIH Image J.

### Quantitating Nuclei

TRAP-stained multinuclear osteoclasts were labeled with DAPI to visualize nuclei. Images of cells were captured with light and fluorescence microscopy and total nuclei were quantitated with NIH Image J. To calculate number of nuclei per cell, DAPI images were overlaid with TRAP stained images to calculate number of nuclei per cell in TRAP positive cells containing 3 or more nuclei.

### Resorption Assay

Primary bone marrow macrophages were plated on Osteo Assay Surface plates (Corning) at a density of 1 × 10^5^ cells per well. Cells were allowed to fully differentiate in the presence of 1.0% CMG 14–12 conditioned media and RANKL. The medium was completely removed on day 5 and 100 μL/well of 10% bleach or TRAP stain was added and allowed to incubate at room temperature for 5 minutes. The bleach solution or TRAP solution was then aspirated and the wells were washed twice with 150 μL of dH_2_O. The plate was then allowed to air dry completely at room temperature for 3–5 hours. The wells were observed under 4x objective for the formation of resorption pits and images were captured with light microscopy. Images were measured and analyzed using NIH Image J.

### Fusion Index

Primary osteoclasts were fixed with 4% paraformaldehyde (PFA), TRAP and DAPI stained on day three after RANKL stimulation. To estimate the degree of cell fusion, total number of nuclei and nuclei within multinucleated giant cells (≥3 nuclei/cell) were counted. The fusion index was calculated using the formula fusion index (%) = total number of nuclei within giant cells/total number of nuclei counted × 100.

### Adhesion Assay

Untreated tissue culture plates were coated with 50 μg vitronectin and allowed to sit at 4 °C for 12 hours. Plates were then placed at room temperature for one hour. Day two pre-osteoclasts that were plated at a density of 2.5 × 10^5^/well (on a 12-well plate) were scraped and transferred to the vitronectin-coated plate (one well to one well). After 30 minutes at room temperature the cells were washed twice with 1xPBS. The cells were then fixed with 4% paraformaldehyde (PFA), TRAP stained, and counted.

### Migration Assay

Primary osteoclast precursors were plated at a density of 2 × 10^5^ cells/well on a 12 well plate and allowed to grow until day 2. On day 2 cells were scraped in 250 μL media. Four wells were combined to provide total of 1 mL. Cells were then counted. 1 × 10^5^ cells/well in 1.0% CMG in osteoclast media were plated in the upper chamber. 700 μL of osteoclast media was placed in the lower chamber. The cells were allowed to settle for 2 hours. 1.0% CMG stimulant was then added to the lower chamber and the cells were allowed to migrate for 18 hours. Cells were then removed from the upper chamber using a cotton swab. Cells on the bottom of the filter and in the lower chamber were fixed using 4% paraformaldehyde (PFA) for 10 minutes. The fixed cells were then stained with TRAP and counted.

### Tunneling Nanotube observation

Primary osteoclasts were fixed with 4% paraformaldehyde (PFA) and washed with PBS. The cells were then stained for TRAP on day 3 after RANKL treatment and imaged under the 4x objective. Three images were taken per well for each well on a 12 well plate. Twelve images per group were calculated. A tunneling nanotube was determined to be present and counted when there was a long extension connecting two cells. The experiment was completed in triplicate.

### Immunofluorescence

Cells were grown on glass coverslips, fixed in picric acid/formaldehyde for 10 minutes, washed with PBS/glycine three times, and then placed in 70% ethanol for 10 minutes. Rhodamine phalloidin (Invitrogen) was added to the cells at 1:40 and placed in the dark for one hour. After five washes of PBS/glycine, cells were stained with NucBlue® (Life Technologies), washed, and mounted with 90% glycerol. Images were obtained using an Olympus Fluoview 500 confocal microscopy and processed using Adobe Photoshop. Individual slices of images are shown.

### Statistics

All experiments were completed in triplicate and performed at least three times. The data shown are representative of the mean ± SD of all experiments. Student’s t-test or 1-way ANOVA analysis followed by a Tukey’s multiple comparison test were used to compare data; p < 0.05 indicates significance. Statistical analysis was performed using Prism 5 software for Mac OSX.

### Data availability Statement

No datasets were generated or analyzed during the current study.

## Electronic supplementary material

Supplementary Information
