# Supplementary material for: Regulation of Osteoclast Differentiation by Myosin X
Source: Sci Rep. 2017 Aug 8;7:7603. doi: 10.1038/s41598-017-07855-9 (PMC5548914; doi:10.1038/s41598-017-07855-9)
Supplement: Supplementary file 1 — Supplementary Information [file 41598_2017_7855_MOESM1_ESM.pdf]

Regulation of Osteoclast Differentiation by Myosin X

Amy Tasca[1], Kristina Astleford[3], Ari Lederman[1], Eric D. Jensen[1], Beth S. Lee[2], Rajaram Gopalakrishnan[1] and Kim C. Mansky[3*]

A.

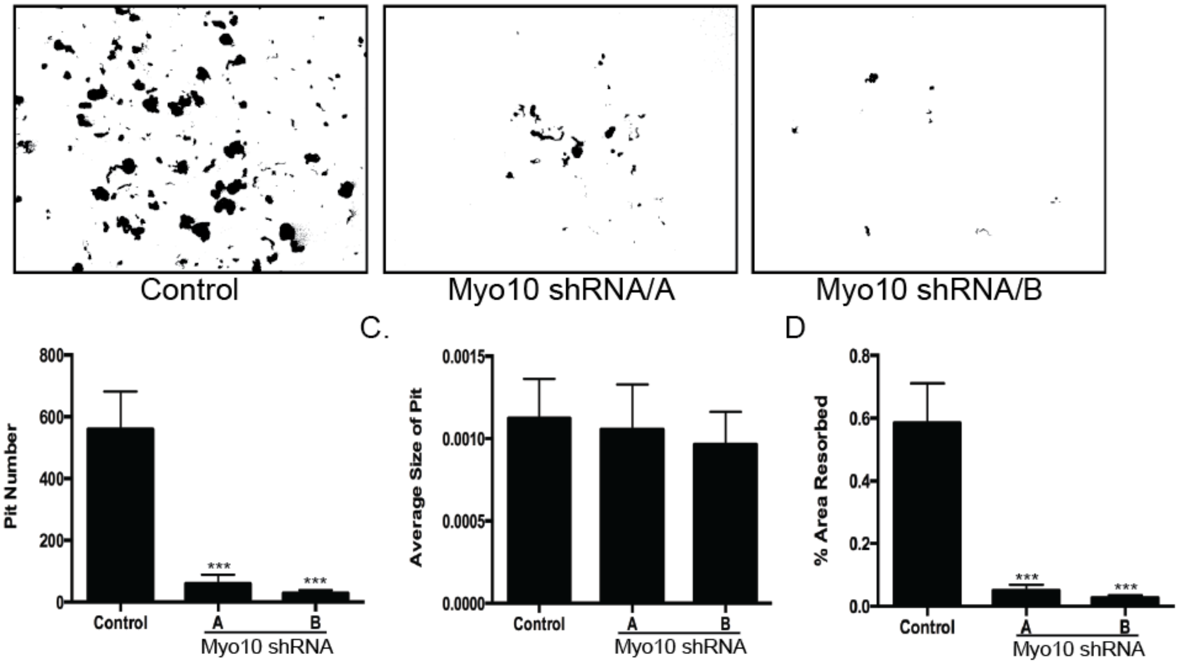

Supplemental Figure 1. *Resorption is reduced when Myo10 expression is reduced in osteoclasts*. BMMs were flushed from C57Bl/6 mice and infected with lentivirus expressing either a control shRNA or *Myo10* targeting shRNA and plated on calcium phosphate coated plates in the presence of M-CSF and RANKL. (A) Representative images of calcium phosphate coated wells. Quantification of (B) number of pits, (C) average area of pits, (D) percent area resorbed. Experiments were done at least three times and values represent the mean ± SD. *** $p<0.001$

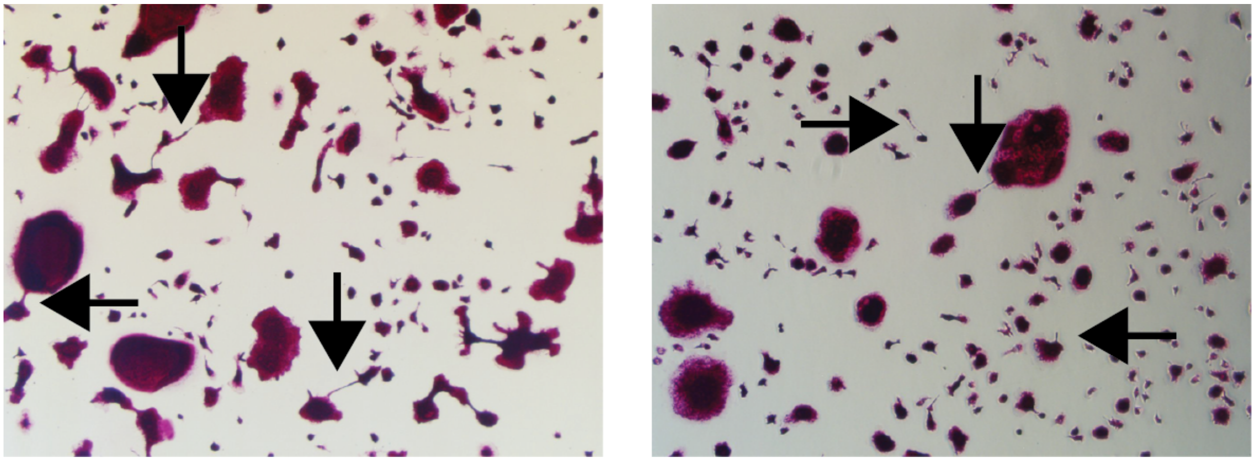

Supplemental Figure 2. *Images of tunneling nanotubes in osteoclasts*. Additional images of tunneling nanotubes in TRAP positive multinuclear osteoclasts. Arrows indicate presence of tunneling nanotubes.

A.

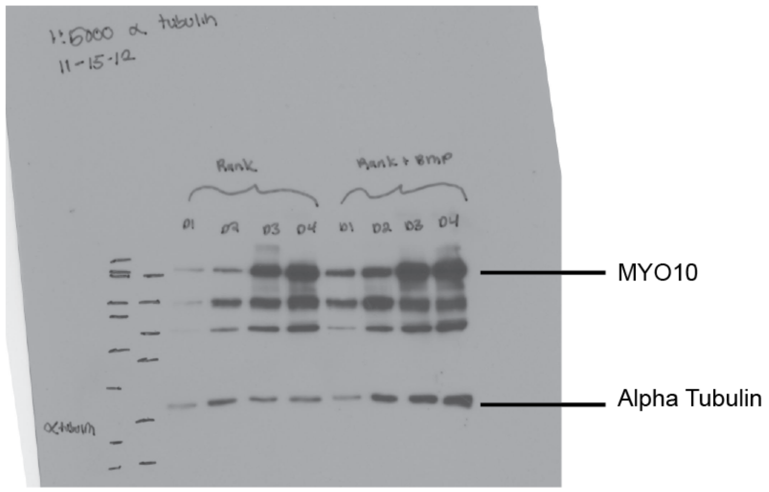

B.

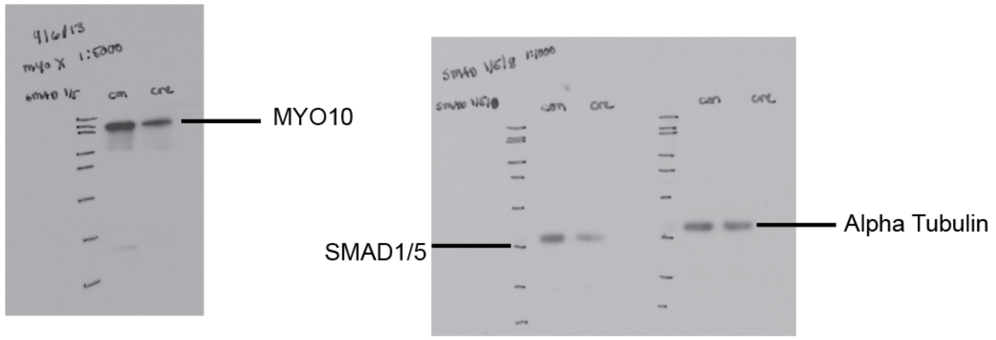

C.

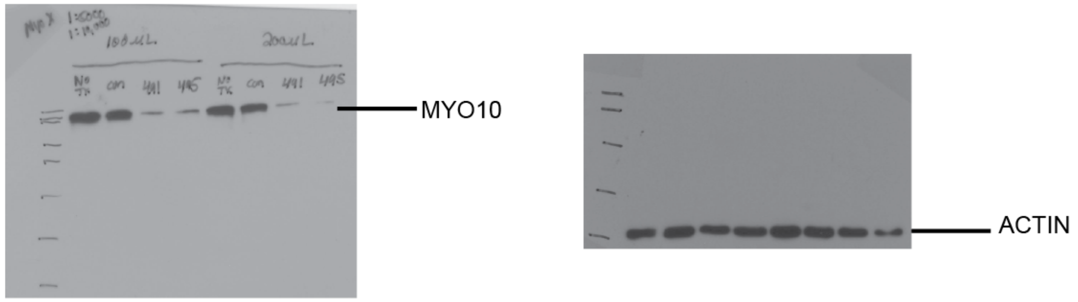

Supplemental Figure 3. *Full length western blots from Figure 1*. Labels beside the blot indicate the protein that was detected by the antibody used in the western blot. (A) Full length blot shown in figure 1A. (B) Full length blots shown in figure 1B. (C) Full length blots shown in figure 1D. In Figure 1D we only showed control and 491 and 495 samples since the no treatment lane was the same as the control. Additionally, we only showed the lower amount of virus (100 ul) lane since both amounts of virus showed a similar pattern.

A.

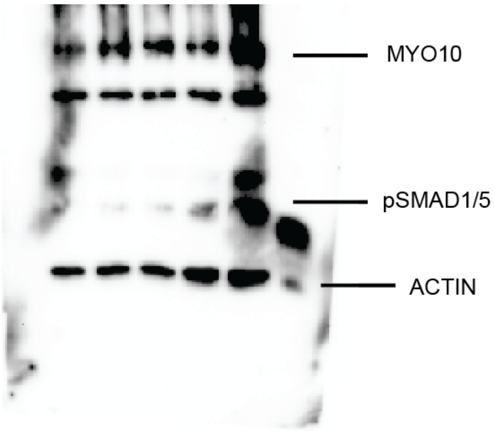

B.

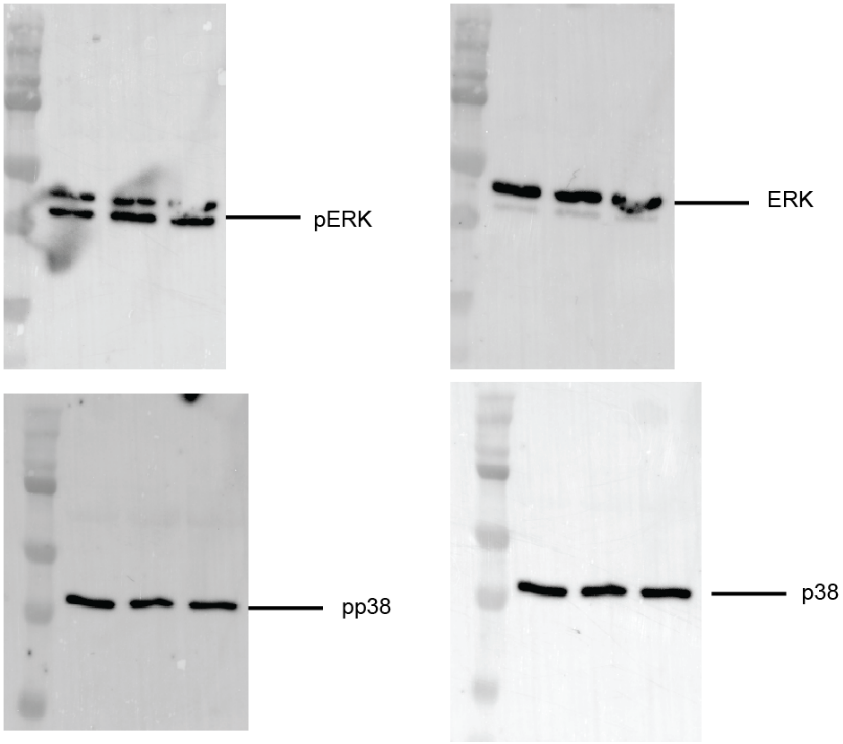

Supplemental Figure 4. *Full length western blots from Figure 4*. Labels beside the blot indicate the protein that was detected by the antibody used in the western blot (A) Full length western blot shown in figure 4A except for the NFATc1 blot. Going from right to left the lanes are control, 491 (100 ul), 495 (100 ul), 491(200 ul), and 495 (200 ul). Again in figure 4A shown in the text we only presented control and the lanes with the lower amount of Myo10 shRNA (491 and 495) (B) full length western blots shown in figure 4B.
